# Normal color variations of the canine ocular fundus, a retrospective study in Swedish dogs

**DOI:** 10.1186/1751-0147-53-13

**Published:** 2011-02-25

**Authors:** Marie IKS Granar, Bo R Nilsson, Helene L Hamberg-Nyström

**Affiliations:** 1 Falun Small Animal Hospital, Samuelsdalsvägen 2B, S-791 61 Falun, Sweden; 2 Cancer Epidemiology Unit, Radiumhemmet, Karolinska Institutet, S-171 76 Stockholm, Sweden; 3 Department of Ophthalmology, St Erik's Eye Hospital, Karolinska Institutet, S-112 82 Stockholm, Sweden

## Abstract

**Background:**

A retrospective study was made to demonstrate normal variations of the color and size of the tapetal area and color of the nontapetal area in the ocular fundus in dogs, correlating them to breed, age and coat color.

**Methods:**

The study was based on protocols of five hundred and thirty-nine adult dogs describing eye examinations made during the years 1997-2001. The dogs were examined using an indirect ophthalmoscope in order to find heritable eye diseases. The following characteristics were recorded: breed; age; coat color; color and size of the tapetal area and color of the nontapetal area. Normal color variations in the fundus were studied and categorized with regard to breed, age and coat color. Chi-square analysis was used comparing distributions between factors. Differences between mean values were analysed with Student's t-test or one-way-ANOVA. A logistic regression analysis was performed on the color of the tapetal area with the color of the coat and breed.

**Results:**

Twenty breeds were represented. The mean age was 42.8 months. The most common colors of the tapetal area were yellow-green and orange, and the most common colors of the nontapetal area were dark brown and black. The analysis revealed that coat-color and breed concomitantly did not significantly influence tapetal color. Brown coated dogs often had a striped red and brown nontapetal area. The color of the tapetal area influenced the color of the nontapetal area. Smaller-sized breeds (such as Papillon) had a smaller tapetal area. A tapetal area was completely absent in 1.9%. The age did not influence the color of the tapetal area.

**Conclusions:**

Color of the tapetal area was influenced by both coat color and breed, but neither of these was statistically more influential than the other. The color of the tapetal area influenced the color of the nontapetal area. The size of the tapetal area correlated to breed and to body size.

## Background

The posterior part of the eye visualized with an ophthalmoscope is called the fundus. The retina and the choroid make the appearance of the fundus. Closest to the vitreous is the retina with its pigmented epithelium (RPE). The neuroretina is mostly invisible, but the cells of the RPE are often densely pigmented. The choroid with its large amount of vessels consists of four principal layers and the amount of pigmentation is individual. It is located behind the RPE. In the tapetal area of the fundus there are cells in one of the layers of the choroid that contains reflectile rodlets. The part of the RPE that overlies the tapetal area is unpigmented and thus makes it possible to see the color of these tapetal cells. A layer of the choroid, the medium sized layer, is commonly heavily pigmented. The appearance of the fundus is due to the pigmentation in all of these layers [1-3]. The tapetal area is located in the dorsal part of the fundus and covers, when full sized, about one third of the fundus [2].

Much of the available literature describing canine eyes concerns pathological changes compared with the normal state. Over the past 50 years there are few publications describing the normal eye. Wyman et al. described the findings of the ocular fundus of the normal dog in 1965 [1]. The appearance of the fundus varies with breed, age and coat color within what is considered normal limits. In the retina about twenty arterioli radiating from the optic disc and 3-4 major veins are seen. The choroid has a lot of vessels appearing as regular striations when the RPE is unpigmented [2]. The tapetal area and the nontapetal area can differ in color, size and shape [1-6]. The color of the tapetal area can vary from blue to gray in puppies and to green, yellow and orange in adult dogs [1,2]. All or parts of the fundus can be depigmented in dogs with a subalbinotic fundus so that the rather straight choroidal vessels are visible with the white sclera underlying [2,7]. Sometimes the tapetal area is very small and may even appear as small spots [2]. It can also be totally absent, which is the case in certain Labrador Retriever families [2,4]. The size of the tapetal area varies considerably and is often breed-linked. The Miniature Poodle and the Papillon can, for example, have a very small tapetal area [4].

The objective of this study was to describe the normal variation in color and size of the tapetal area and color of the nontapetal area with regard to breed, age and coat color. The most common color of the tapetal area was yellow-green and the most common color of the nontapetal area was dark brown. Brown coated dogs had a more orange-tinted tapetal area and a redder nontapetal area. A tapetal area was missing in 1.9% of the animals examined. Smaller-sized breeds had significantly smaller size of the tapetal area. Dogs with a green or blue-green tapetal area also had a smaller size of this area. Color of the tapetal area was determined by both coat color and breed, but neither of these points was statistically more influential than the other.

## Methods

This is a retrospective study based on protocols describing eye examinations made during the years 1997 - 2001. The examinations were made by the first author at Falun Small Animal Hospital, Mora District Veterinary Station and Väsby Veterinary Clinic. Examination of 539 adult dogs was conducted using an indirect ophthalmoscope (Heine HK 4000, Heine Optotechnik GmbH & Co, Herrsching, Germany) together with a clear 20 diopter lens. The reason for the visit for all dogs was to be examined for heritable eye diseases. Both eyes were examined. The dogs were given a drop of 0.5% tropicamide (Mydriacyl^®^, Alcon, Stockholm, Sweden) in each eye at least 20 minutes before being examined in a darkened room. None of the dogs were sedated.

**Figure 1:**
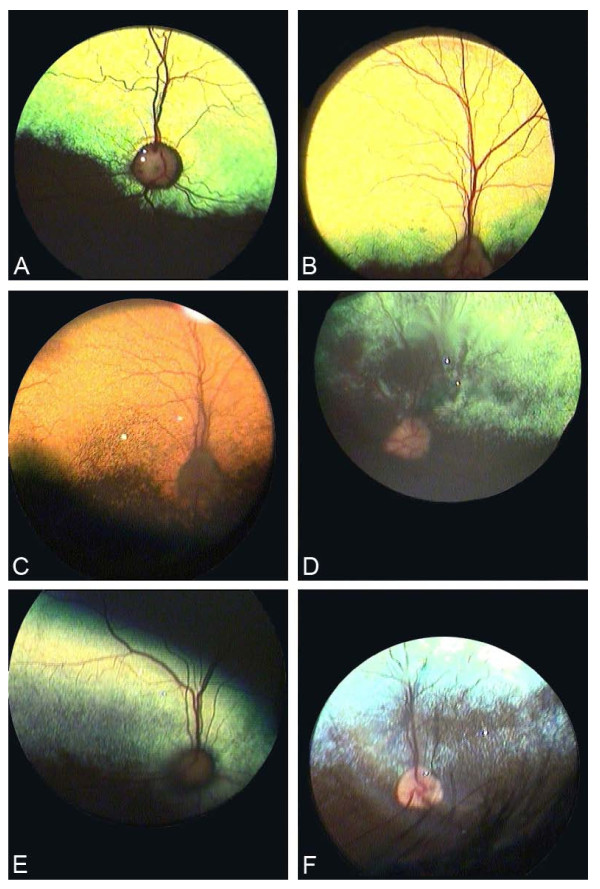
**Examples of different colors of the tapetal area in the dog **A: Yellow-green, B: Yellow, C: Orange, D: Green, E: Green, F: Blue-green

The animals' breed, age, coat color, color and size of the tapetal area and color of the nontapetal area were noted. Breeds were classified according to the Swedish Kennel Club classification of group and breed. Only breeds with ten or more dogs were included. Age was presented in months. All dogs were 10 month or more. Coat color was divided into white, gray (including wild type color and dappled grey), yellow, red, brown, black, tricolor, sable and merle. The dominant color determined to which group the dog was assigned. Ocular observations were made of both right and left eyes, but only the right eye was used for describing colors and size of the tapetal area and color of the nontapetal area. The measurement of size and description of colors were made by subjective evaluation with the following criteria:

The color of the tapetal area was divided into blue-green, green, yellow-green, yellow and orange. The color of the tapetal area in dog can have a mix of colors but also here the dominant color determined to which group the dog was assigned. Figure 1 shows some of the colors. The photographs were made with an indirect ophthalmoscope (Heine video Omega 2 C with an A-cam camera, Heine Optotechnik GmbH & Co, Herrsching, Germany). Total absence of tapetal area was also noted.

The color of the nontapetal area was divided into black, dark brown, brown, red-brown, red, striped red and brown and subalbinotic (striped red and white).

**Figure 2:**
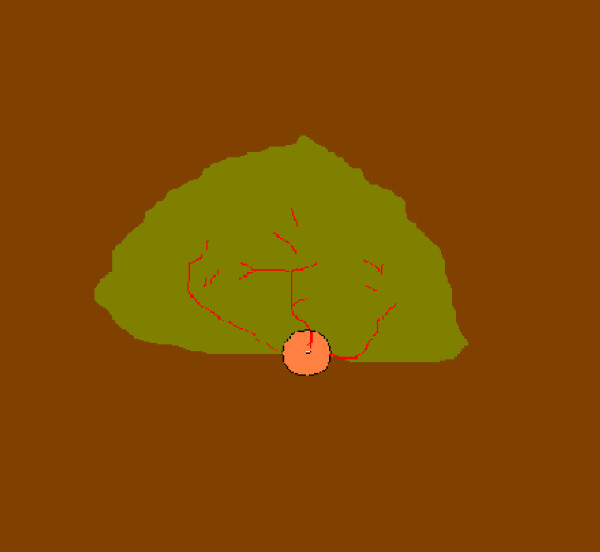
**A schematic drawing of the fundus with a full sized tapetal area **The tapetal area forms an almost triangular area, with a horizontal base, in the dorsal half of the fundus. When estimated full sized it covers about one third of the fundus.

The size of the tapetal area was set at 100% if considered to be full sized covering about one third of the dorsal fundus with the optic nerve situated near the border between tapetal and the nontapetal area as shown in Figure 2. Lesser area was estimated as per cent of full size.

### Statistical methods

Chi-square analysis was used comparing distributions between factors. Differences in mean was analysed with Student's t-test or one-way-ANOVA. A logistic regression analysis was performed on the color of the tapetal area with the color of the coat and breed.

## Results

### Breed, age and coat color

**Figure 3:**
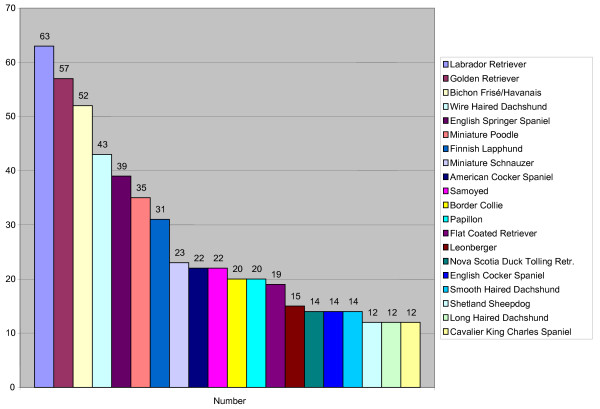
**Total number of eye-examinations in different breeds**.

Five hundred thirty-nine dogs in twenty different breeds were included in the study. The most prevalent breeds were Labrador Retriever (N = 63), Golden Retriever (N = 57) and Bichon Frisé/Havanais (N = 52). The distribution of breeds is shown in Figure 3. The mean age was 42.8 months ± SD, with a median age of 38.0 months. Black (33.6%) was the most dominant coat color followed by yellow (20.4%), white (13.4%), brown (11.7), gray (10.8%), tricolor (5.2%), red (3.5%) sable (0.9%) and merle (0.5%).

### The color of the tapetal area

**Figure 4:**
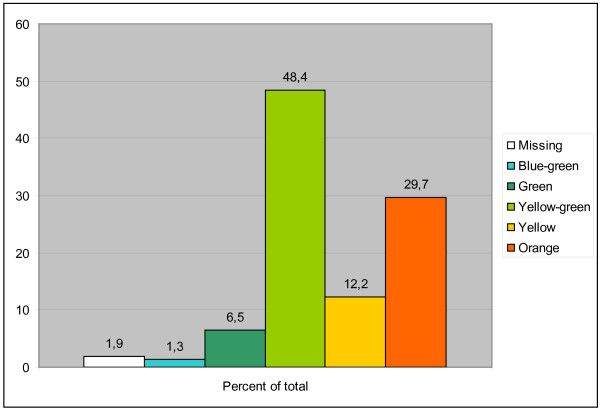
**Frequencies of different colors of the tapetal area**.

Colors of the tapetal area were categorized as in Figure 4. Yellow-green was the most common color (48.4%, N = 261), followed by orange (29.7%, N = 160) and yellow (12.2%, N = 66). The tapetal area was absent in 1.9% (N = 10) of the animals examined. The color of the tapetal area was different in the right and left eye in 9 of 539 dogs. The distribution of colors of the tapetal area in the eight most common breeds is shown in Table 1. The color distribution varied quite considerably between breeds. The Miniature Schnauzer, for example, had a high amount of green and blue-green tapetal area and the English Springer Spaniel had a high percent of orange tapetal area. Most of the other breeds had a predominantly yellow-green tapetal area. The age did not influence the color of the tapetal area.

**Table 1:** Color of the tapetal area

Color	Missing	Blue- green	Green	Yellow- green	Yellow	Orange
Breed						
Labrador Retriever N = 63	4.8%	1.6%	1.6%	54.0%	36.4%	1.6%

Golden Retriever N = 57				45.6%	24.6%	29.8%

Bichon Frisé./Hav N = 52			5.8%	61.5%		32.7%

Wirehaired Dachshund N = 43		2.3%	23.3%	67.4%	7.0%	

English Springer Spaniel N = 39				51.3%	2.6%	46.1%

Miniature Poodle N = 35			5.7%	54.3%	11.4%	28.6%

Finnish Lapphund N = 31				58.1%	12.9%	29.0%

Miniature Schnauzer N = 23		13.0%	43.5%	43.5%		

(Percent within breed)

In the eight most common breeds

The relationship between coat and color of the tapetal area is shown in Table 2 and indicate that dogs with brown and red coat colors had a more orange-tinted tapetal color. The dogs that had a green-colored tapetal area often had white or grey coat.

**Table 2:** The distribution of color of the tapetal area compared to coat color

Color of the tapetal area	Missing	Blue- green	Green	Yellow- green	Yellow	Orange
Coat Color						
White N = 72	2.8%	1.4%	9.7%	54.2%	1.4%	30.5%

Grey N = 58		1.7%	20.7%	58.6%	6.9%	12.1%

Yellow N = 110	0.9%	0.9%	1.8%	40.9%	27.3%	28.2%

Red N = 18				27.8%	5.6%	66.6%

Brown N = 63			3.2%	44.4%	8.0%	44.4%

Black N = 181	1.1%	1.7%	6.6%	54.7%	10.5%	25.4%

Tricolor N = 28	14.3%			35.7%	10.7%	39.3%

Sobel N = 5				20.0%	40.0%	40.0%

Merle N = 3	66.7%			33.3%		

Percent within different coat colors

A logistic regression analysis was performed on the color of the tapetal area with the color of the coat and breed. Only the four most common breeds (Labrador retriever, Golden Retriever, Bichon Frisé/Havanais and Wire Haired Dachshund) were included, as independent factors. The analysis revealed that coat-color and breed concomitantly did not significantly influence tapetal color.

### The color of the nontapetal area

**Figure 5:**
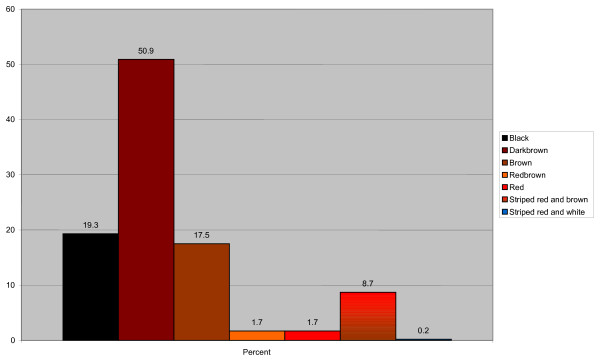
**Frequencies of different colors of the nontapetal area**.

The distribution of colors of the nontapetal area is shown in Figure 5. The most common color was dark brown (50.9%, N = 273), followed by black (19.3%, N = 104) and brown (17.5%, N = 94). Color of the nontapetal area in the five most common breeds is shown in Table 3. The color distribution varied quite considerably between breeds. Golden Retrievers had a large proportion of black nontapetal area. The English Springer Spaniel, that often had a brown coat color, had a high proportion of striped red and brown nontapetal area (51.3% of all in this breed) often combined with an orange color of the tapetal area. The other breeds had a high frequency of dark brown. The age did not influence the color of the nontapetal area.

**Table 3:** The color of the nontapetal area

Color of nontapetal area	Black	Dark brown	Brown	Red- brown	Red	Striped red and brown	Striped red and white
Breed							
Labrador Retriever N = 63	23.8%	61.9%	12.7%		1.6%		
Golden Retriever N = 57	42.1%	42.1%	15.8%				
Bichon Frisé/Hav. N = 52	17.3%	67.3%	15.4%				
Wirehaired Dachshund N = 43	11.6%	60.5%	27.9%				
English Springer Spaniel N = 34	2.5%	25.6%	7.7%	2.6%	10.3%	51.3%	

Percent within breed

In the five most common breeds

A comparison between colors of the tapetal area and the nontapetal area is shown in Table 4. The color of the tapetal and the nontapetal area had a high degree of correlation. The dogs with an orange tapetal area often had a dark pigmented nontapetal area such as black or dark brown but some of them had a striped red and brown nontapetal area. The individuals with a yellow tapetal area had an even more pigmented nontapetal area with 46.2% of the dogs having a black color. The most common combination was a yellow-green tapetal area and dark brown nontapetal area (41.6%, N = 224).

**Table 4:** The relationship between the color of the tapetal area and the color of the nontapetal area

Nontapetal area	Black	Dark brown	Brown	Red- brown	Red	Striped red and brown	Striped red and white
Tapetal area							
Missing N = 10		40.0%	40.0%			20.0%	

Blue- Green N = 7	14.4%	42.8%	42.8%				

Green N = 35	5.7%	60.0%	34.3%				

Yellow- green N = 262	7.7%	63.8%	15.7%	1.2%	0.5%	11.1%	

Yellow N = 65	46.2%	33.8%	10.8%	4.6%	3.1%	1.5%	

Orange N = 160	31.9%	36.3%	16.9%	1.9%	3.7%	8.7%	0.6%

Percent within the category of the color of the tapetal area

The coat color compared to the color of the nontapetal area is shown in Table 5. Dogs with yellow coat had the highest percent of black nontapetal area. If the coat color was red or brown the nontapetal area had a high incidence of striped red and brown color.

**Table 5:** Distribution of the color of the nontapetal area compared to coat color

Color of the nontapetal area	Black	Dark brown	Brown	Red- brown	Red	Striped red and brown	Striped red and white
Coat color							
White N = 72	8.3%	61.2%	18.1%	2.8%	2.8%	6.8%	

Grey N = 58	10.3%	63.8%	25.9%		0%	0%	0%

Yellow N = 110	35.5%	50.0%	13.6%			0.9%	

Red N = 18	16.7%	38.9%	11.1%		5.5%	27.8%	

Brown N = 63	11.1%	19.1%	7.9%	6.4%	9.5%	46.0%	

Black N = 181	18.8%	56.9%	21.5%	1.7%		1.1%	

Tricolor N = 28	21.4%	50.0%	17.9%			7.1%	3.6%

Sobel N = 5	40.0%	40.0%				20.0%	

Merle N = 3	33.3%					66.6%	

Percent within coat color

### The size of the tapetal area

**Figure 6:**
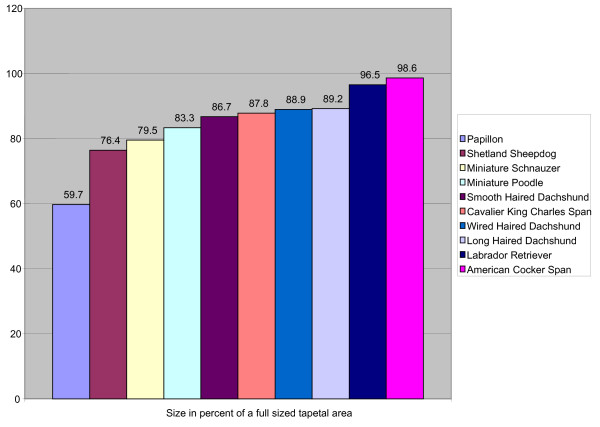
**Size of the tapetal area in different breeds**. The size of the tapetal area is judged as 100% when considered full sized.

The tapetal area was full sized in 70.3% of the examined dogs and this area was entirely absent in 1.9%. The size in some breeds is seen in Figure 6. The Papillon was the breed with the smallest size of the tapetal area. Dogs with a full-sized tapetal area included Border Collie, Leonberger, Samoyed, Golden Retriever and English Springer Spaniel. Labrador Retrievers had smaller than expected mean tapetal size because a fairly large proportion of this group lacked the tapetal area altogether (4.8% within the Labrador Retriever group). This study shows that smaller-sized breeds had significantly smaller tapetal area. Breeds with smaller body size and with a average weight below 10 kg (Shetland Sheepdog, Dachshunds, American Cocker Spaniel, Miniature Schnauzer, Miniature Poodle, Bichon Frisé/Havanais, Cavalier King Charles Spaniel and Papillon) had a tapetal size mean of 88.0% (SD ± 21.5, n = 217) compared to the rest (larger sized breeds) had a mean of 99.1% (SD ± 9.0, n = 244) p < 0.001, Student's-t-test).

The color of the coat influenced the size of the tapetal area (p < 0.001, ANOVA test for heterogeneity). The dogs with a grey coat color had a smaller sized tapetal area.

The color of the tapetal area showed a strong correlation with the size of the tapetal area (p < 0.001, ANOVA test for heterogeneity). Dogs with a green or blue-green tapetal area had smaller sized tapetal area (mean = 84.6%, SD ± 16.8, n = 37) compared with other colors (mean = 96.5, SD ± 10.8, n = 415) (p < 0.001).

## Discussion

In this study the most common color of the tapetal area was yellow-green and the most common color in the nontapetal area was dark brown. Both breed and coat color influenced the color of the tapetal area. Whether one or the other had more influence could not be established. Brown-coated dogs had a more orange tinted tapetal area and the nontapetal area tended to be redder. The tapetal area was absent in 1.9%. Size of the tapetal area depended more on the breed and its body size than on coat color.

### The tapetal area

The tapetal cells is believed to increase retinal light sensitivity by reflecting light back and thereby give the retina two chances to react to the light [8-10]. This phenomenon may make the image more blurred. It has not been made clear whether dogs that either lack tapetal area in the fundus or are subalbinotic have poorer vision overall or poorer night vision compared to dogs with tapetal area [11]. This study found that 1.9% lacked tapetal area and that the size can vary, but the degree of sight has not been examined here. The tapetal area in dogs can vary considerably in size and the color can vary from blue-green to yellow-green [1-3,5,6]. In this study yellow-green was the most common color followed by orange and yellow.

Histologically the part of the choroid that gives the tapetal area its appearance in dogs consists of 15-20 layers of tapetal cells in the central area. Towards the periphery it gradually thins and eventually ends. The tapetal cells are tightly packed with bundles of rodlets. The tapetal cells in animals can contain guanine, hypoxanthine and riboflavin [9]. Zinc and cystein are the major chemical substances present in the tapetal cells in dogs [10,12]. It is not completely clear what makes the difference in the colors but both the thickness of the layer of tapetal cells and chemistry in these cells influences wavelength and the amount of light reflected by this part of the eye [8-10]. Young puppies exhibit a strong bluish reflection from their tapetal area [12]. The earliest color of the tapetal area in young puppies is purple, then turns into pale green and by the age of 2 -3 months into the adult appearance [1,13]. No puppies were included in this study. It has been debated about the association between breed and color of the tapetal area [2,4,13]. In this study both coat color and breed influenced colors of the tapetal area but no statistical difference between these two factors was found.

### The nontapetal area

The nontapetal area of the fundus may also vary considerably in color, from almost black to brown, red and striped [2,7]. Coloring of this part depends on the degree of pigmentation in the choroid [1,2] and on the pigmentation in RPE [2]. When RPE lacks pigmentation the nontapetal area is often striped red and brown as you can see the straight choroidal vessels in the brownpigmented choroid. If also the choroid is lacking pigmentation, the white sclera beneath can be seen with the overlying choroid vessels and the nontapetal area now looks striped red and white [2]. The most common color of the nontapetal area in this study was dark brown, followed by black. In this study the color of the nontapetal area was influenced by both breed and coat color. It also correlated with colors of the tapetal area, for example a yellow tapetal area often had a black nontapetal area and dogs with orange and yellow-green tapetal area had a high percent of striped red and brown nontapetal area.

### The influence of the coat color

The tapetal area in brown coated dogs often shows orange-tinted color. The nontapetal area in these dogs has been reported to be red-brown or striped red and brown in color [2,14] and is in agreement with our findings. The fundus in Samoyed dogs may vary in color and appearance although the coat color is always white [4]. In this study, the color of the tapetal area of the 22 adult Samoyeds ranged between blue-green (4.5%), green (18.2%), yellow-green (54.6%) and orange (22.7%).

### The size of the tapetal area

The tapetal area of the fundus covers a triangular area of approximately 30% of the superior fundus [1,3,8,9]. The optic nerve head most commonly lies in the border between the tapetal and the nontapetal area [6]. Certain breeds have a large tapetal area that surrounds the optic nerve head while some smaller-sized breeds, like the Papillon, have a small tapetal area [2,7,14,15]. The tapetal area in large dogs is often large [2,7,14]. This study supports the fact that smaller sized breeds have a smaller tapetal area but no giant breeds were included in these observations. The tapetal area in Miniature Poodles may vary considerably in size and distribution, and can sometimes be present as islands in the nontapetal area [4,16]. In this study, both Miniature Poodles and Papillons had a relatively small tapetal area. Some dogs lack a tapetal area altogether [2]. Labrador Retrievers can miss the tapetal area in certain families [2,4] and this study found that 4.8% out of 63 Labradors lacked this structure.

### Limitations of the study

The study was made in the middle part of Sweden so it represents the normal variation in the population of dogs from this part of the country. The dogs included in the study were of breeds where eye examinations are common and the results might be different if a randomly selected material had been chosen. All factors including colors and sizes in the eye were judged by impression of the examiner. Prior to papillary dilation, no iridal observations were made with regard to their color. Thus, as a consequence of tropicamid drops, it was impossible to judge the color of the iris.

## Conclusion

The most common color of the tapetal area was yellow-green and the most common color of the nontapetal area was dark brown. Brown coated dogs had a more orange-tinted tapetal area and a redder nontapetal area. This study could not establish whether breed or coat color had most influence on the color of the fundus. A small size of the tapetal area was seen in breeds with small body size. The tapetal area was absent in 1.9% of the animals examined.

## Declaration of competing interests

The authors declare that they have no competing interests.

## Authors' contributions

MIKSG designed and coordinated the study, collected the data and had the responsibility for writing and finalising the manuscript.

BRN participated in the design of the study and performed the statistical analysis.

HLHN conceived of the study, and participated in its design and coordination and helped to draft the manuscript.

All authors read and approved the final manuscript.
